# Recent Advances in Producing Sugar Alcohols and Functional Sugars by Engineering *Yarrowia lipolytica*

**DOI:** 10.3389/fbioe.2021.648382

**Published:** 2021-03-11

**Authors:** Abdul Rahman Abbasi, Jinle Liu, Zhi Wang, Anqi Zhao, Hanjie Ying, Lingbo Qu, Md. Asraful Alam, Wenlong Xiong, Jingliang Xu, Yongkun Lv

**Affiliations:** ^1^School of Chemical Engineering, Zhengzhou University, Zhengzhou, China; ^2^School of Agricultural Sciences, Zhengzhou University, Zhengzhou, China; ^3^School of Life Sciences, Zhengzhou University, Zhengzhou, China; ^4^Zhengzhou Tuoyang Industrial Co., Ltd., Zhengzhou, China; ^5^Zhengzhou University Industrial Technology Research Institute Co., Ltd., Zhengzhou, China; ^6^Key Laboratory of Advanced Drug Preparation Technologies, Ministry of Education, School of Pharmaceutical Sciences, Zhengzhou University, Zhengzhou, China

**Keywords:** sugar alcohols, functional sugars, *Yarrowia lipolytica*, gene discovery, substrate scope, strain breeding

## Abstract

The sugar alcohols and functional sugars have wide applications in food, pharmaceutical, and chemical industries. However, the smaller quantities of natural occurring sugar alcohols and functional sugars restricted their applications. The enzymatic and whole-cell catalyst production is emerging as the predominant alternatives. The properties of *Yarrowia lipolytica* make it a promising sugar alcohol and functional sugar producer. However, there are still some issues to be resolved. As there exist reviews about the chemical structures, physicochemical properties, biological functions, applications, and biosynthesis of sugar alcohols and/or functional sugars in *Y. lipolytica*, this mini review will not only update the recent advances in enzymatic and microbial production of sugar alcohols (erythritol, D-threitol, and xylitol) and functional sugars (isomaltulose, trehalose, fructo-oligosaccharides, and galacto-oligosaccharides) by using recombinant *Y. lipolytica* but also focus on the studies of gene discovery, pathway engineering, expanding substrate scope, bioprocess engineering, and novel breeding methods to resolve the aforementioned issues.

## Introduction

Sugar alcohols or polyols are defined as a group of acyclic hydrogenated carbohydrates, but they are not sugars. Functional sugars generally refer to the sugars with unique structural and physiological characteristics, but occur in very low quantity in nature (Bilal et al., 2020). Sugar alcohols and functional sugars have various applications in pharmaceutical, agro-food, and chemical industries (Fickers et al., 2020). With the properties of same or better sweeting but less caloric value of sucrose, sugar alcohols and functional sugars are evolving as food ingredients (Park et al., 2016). Besides, they are increasingly used in pharmaceutical applications because of their excellent functional properties and health benefits. It was estimated that the global consumption of sugar alcohols will reach 1.9 million metric tons by 2022 (Grembecka, 2015). However, sugar alcohols and functional sugars are present in smaller quantities in nature (plants, fungi, and algae). *In planta*, sugar alcohols are temporarily accumulated in leaves during light and are transported to other organs during dark. The low content makes plant extraction of sugar alcohols difficult. Chemical approaches to sugar alcohols and functional sugars suffer from strict reaction conditions, limited yield, expensive raw starting feedstocks, and safety risks (Huang et al., 2018). Engineering enzymes or whole-cell catalyst is emerging as the predominant alternative approach (Fickers et al., 2020).

The non-model organism *Yarrowia lipolytica* is an emerging host for the production of non-native chemicals and is expeditiously becoming a model and unique microorganism with strong industrial potentials (Sharma and Lee, 2020). *Y. lipolytica* was empowered by the recently developed genetic and synthetic biology tools and metabolic engineering methods (Madzak, 2018; Shi et al., 2018; Ganesan et al., 2019). *Y. lipolytica* has been harnessed for heterologous synthesis of many bioactive natural compounds such as polyketides, isoprenoids, α-santalene, limonene, (+)-nootkatone, protopanaxadiol, gensenoside K, astaxanthin, and flavonoids (Cao et al., 2016; Guo et al., 2018; Jia et al., 2019; Li et al., 2019; Lv et al., 2019; Tramontin et al., 2019; Wu et al., 2019; Luo et al., 2020; Palmer et al., 2020).

Promising results have been achieved by engineering *Y. lipolytica* into efficient producer of sugar alcohols and functional sugars due to its low nutritive requirements, feasibility of high cell density culture, and ease of genome editing (Fickers et al., 2020). However, there remain some issues (inefficiency of enzyme activity and microbial cell factory, high cost of substrates, suboptimal bioprocess, and strain unstability) to be resolved before making the bioprocesses commercially feasible. As the physicochemical properties, biological functions, applications, and biosynthesis of sugar alcohols and functional sugars have been discussed in multiple reviews (Grembecka, 2015; Carly and Fickers, 2018; Bilal et al., 2020; Fickers et al., 2020), this mini review will update the recent advances (2016–2020) in enzymatic and microbial production of sugar alcohols (erythritol, D-threitol, and xylitol) and functional sugars (isomaltulose, trehalose, fructo-oligosaccharides, and galacto-oligosaccharides) by using recombinant *Y. lipolytica* (Table 1) and focus on the studies of gene discovery, pathway engineering, expanding substrate scope, bioprocess engineering, and novel breeding methods to resolve the aforementioned issues.

**TABLE 1:** Sugar alcohols and functional sugars produced by engineering *Y. lipolytica.*

Products	Carbon source	Genetic engineering strategy	Cultivation Strategy	Titer	References
	Glycerol	The overexpressed codon optimized bacterial hemoglobin from *Vitreoscilla stercoraria* was used	Bioreactor culture	55.75 g/L	Mironczuk et al., 2019
	Glycerol	Overexpressed TKL1 and GUT1, Disruption of EYK1	Bioreactor culture	80.6 g/L	Carly et al., 2017; Carlya et al., 2017
	Glycerol	Ultraviolet (UV) mutagenesis and optimal C:N ratio	Chemostat culture	113.1 g/L	Rakicka et al., 2017a
Erythritol	Glycerol	The overexpression of Erythrose reductase encoding gene YALI0F18590gwas used	Batch culture	44.44 g/L	Janek et al., 2017
	Glycerol	The four genes *ZWF1*, *TAL1*, *GND1*, and *TKL1* were functionally overexpressed	Shake flask experiment	51.09 g/L	Mironczuk et al., 2017
	Glycerol	The disruption of Gene encoding erythrulose kinase YALI0F01606g was used	Batch bioreactor	35.7 g/L	Carly et al., 2017; Carlya et al., 2017
	Glycerol	Span 20 surfactant was added	Fed-batch culture	142 g/L	Rakicka et al., 2017b
D-threitol	Glucose	The overexpression of xylitol dehydrogenase gene (*Ss-XDH*) from *Scheffersomyces stipitis*	Culture broth	112 g/L	Chi et al., 2019
Isomaltulose	Sucrose	Displaying sucrose isomerase encoding gene from *Pantoea dispersa* UQ68J (PdSIase) on the *Y. lipolytica* cell surface	Fermentor	465 g/L	Li et al., 2017
	Sucrose	gene encoding sucrose isomerase (SIase) from *Pantoea dispersa*UQ68J	Fermentor	572.1 g/L	Zhang et al., 2018
Trehalose	Maltose	Displaying encoding trehalose synthase gene (TreS) on the Y. lipolytica cell surface	Bioreactor	219 g/L	Li et al., 2016
Fructo-oligosaccharides	Sucrose	Erythritol producing *Y. lipolytica* cells containing displayed fructosyltransferase	Bioreactor	480 g/L	Zhang et al., 2016
	Inulin	The overexpression of optimized endo-inulinase gene from *Aspergillus niger*was used	Bioreactor	546.6 g/L	Han et al., 2017
Galacto-oligosaccharides	lactose	Displaying β-galactosidase on *Y. liplytica* cell surface	Bioreactor	160 g/L	An et al., 2016

## Production of Sugar Alcohols and Functional Sugars in *Yarrowia lipolytica*

### Erythritol

**FIGURE 1:**
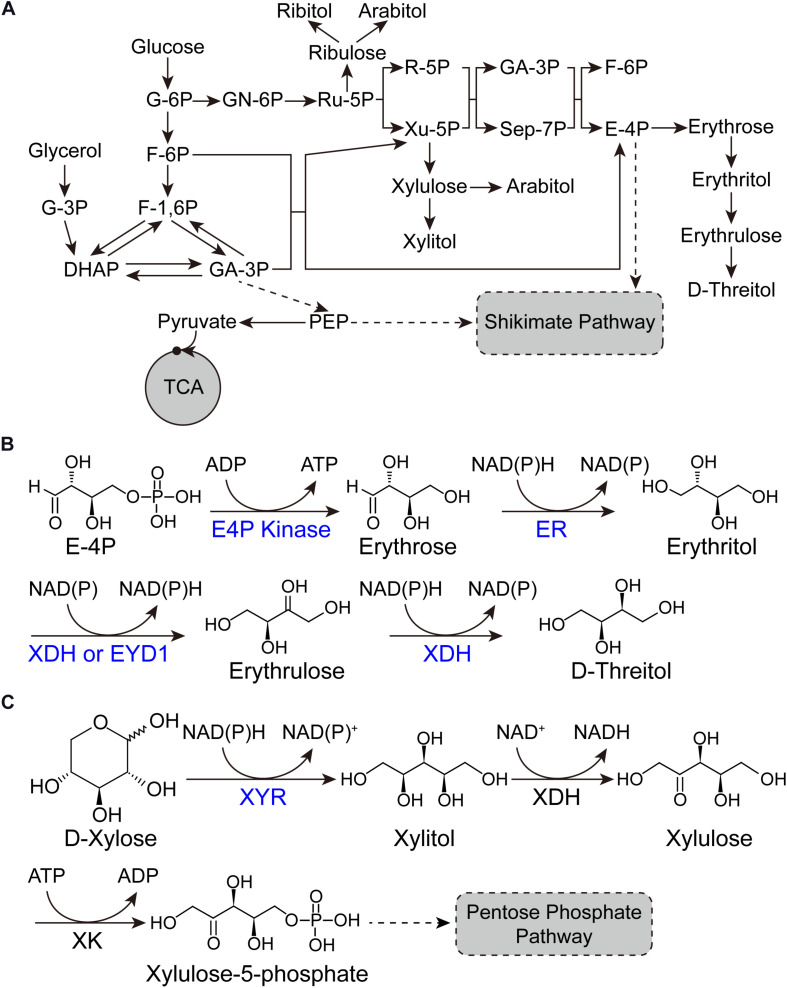
Pathways for sugar alcohol biosynthesis. **(A)** Metabolic pathway of sugar alcohols in *Y. lipolytica*. **(B)** Biotransformation pathway from E-4P to D-threitol. **(C)** Biotransformation pathway from D-xylose to xylitol. G-6P, glucose-6-phosphate; F-6P, fructose-6-phosphate; F-1,6P, fructose-1,6-bisphosphate; DHAP, dihydroxyacetone phosphate; G-3P, glycerol-3-phosphate; GA-3P, glyceraldehyde-3-phosphate; PEP, phosphoenolpyruvic acid; TCA, tricarboxylic acid cycle; GN-6P, gluconolactone-6-phosphate; Ru-5P, ribulose-5-phosphate; R-5P, ribose-5-phosphate; Xu-5P, xylulose-5-phosphate; GA-3P, glyceraldehyde-3-phosphate; Sep-7P, sedoheptulose-7-phosphate; E-4P, erythrose-4-phosphate; ER, erythrose reductase; XDH, xylitol dehydrogenase; EYD1, erythritol dehydrogenase; XYR, xylose reductase; XDH, xylitol dehydrogenase; XK, xylulose kinase.

Erythritol (1,2,3,4-butanetetrol) is a sugar alcohol with four-carbons. Due to its sweetening properties, it is employed as food additive in agro-food industries (Carlya et al., 2017). Erythritol is a safety sweetener for diabetics, because it does not affect the insulin level within the blood due to its chemical features (Janek et al., 2017). It is also used as chemical precursor for the synthesis of substances having phase transition behavior (Qiu et al., 2020). Yeast and bacteria produce erythritol in the form of osmoprotectant. The oleaginous yeast *Y. lipolytica* has been reported for the synthesis of erythritol since 1970s (Mironczuk et al., 2017). *Y. lipolytica* possesses the primary metabolic pathway for erythritol synthesis and can be engineered into erythritol over-producer from both glucose and glycerol by overexpressing erythrose-4-phosphate kinase and erythrose reductase (Figure 1A; Mironczuk et al., 2017). The glycerol is a renewable feedstock and is produced in the form of waste product on large scale in various industries (Mironczuk et al., 2017). Promising results have been achieved in engineering *Y. lipolytica* into erythritol over-producer and expanding its substrate scope, which will be discussed in detail in sections “Gene discovery and pathway engineering” and “Expanding substrate scope and bioprocess engineering” in this mini review.

### D-Threitol

D-threitol is a diastereoisomer of erythritol. Few osmotolerant yeasts synthesize D-threitol as an osmoprotective agent. It is widely used in pharmaceutical, medicine, food, and green chemistry (Carly and Fickers, 2018). D-threitol can be synthesized from erythritol by xylitol dehydrogenase (XDH) with erythrulose as an intermediate, which is the most explored method (Figure 1B). Chi et al. (2019) purified an XDH from *Scheffersomyces stipitis* (Ss-XDH) and found that it has the potential to convert erythritol into erythrulose and threitol *in vitro*. By overexpressing Ss-XDH in an erythritol-producing *Y. lipolytica*, they achieved 112 g/L D-threitol with a yield of 0.37 from glucose (Chi et al., 2019). The upregulation of mannitol dehydrogenase encoding gene resulted in the accumulation of mannitol in the culture broth. They developed the *Candida parapsilosis* culture in order to remove the byproduct (mannitol) and coproduce erythritol to facilitate the subsequent purification of threitol, as *C*. *parapsilosis* has the ability to catabolize erythritol and mannitol but not threitol (Chi et al., 2019).

### Xylitol

Xylitol is a sugar alcohol utilized as sweetener. Naturally, it is present in very small quantities. It has applications in dental and oral diseases. Due to its excellent anti-inflammatory potential, xylitol can be used to cure chronic inflammatory diseases effectively (Benahmed et al., 2020). It is also used to treat the respiratory diseases such as pneumonia and middle ear infections. In mammals, xylitol is responsible for the secretion of insulin in blood plasma. It also plays a role in the reduction of obesity and some other metabolic syndromes (Benahmed et al., 2020). In addition, it is also used in sugar free gum and is commonly not harmful to humans but can be highly toxic to dogs (Rajapaksha et al., 2019). The synthesis of xylitol through chemical process is costly and laborious. On the contrary, xylitol production through microbial cell factories facilitates the inexpensive and profitable alternative process (Prabhu et al., 2020). *Y. lipolytica* possesses the complete xylose utilizing pathway (Figure 1C). However, the expression of xylitol dehydrogenase (XDH) is strictly regulated, resulting in *Y. lipolytica* unable to grow on xylose (Rodriguez et al., 2016). This characteristic has been substantially explored for the bioconversion of xylose into xylitol (Prabhu et al., 2020). For instance, Prabhu et al. established and optimized the bioprocess of converting xylose into xylitol. They found that glycerol is a better co-substrate for biomass accumulation and subsequent bioconversion compared with glucose. After condition optimization and scale up in bioreactor, they achieved 53.2 g/L xylitol from pure glycerol (PG) with a yield of 0.97 g/g. When they substituted PG with biodiesel-derived crude glycerol (CG), similar results (50.5 g/L xylitol with a yield of 0.92 g/g) were achieved. They also tried sugarcane bagasse hydrolysate as the feedstock and achieved 0.54 g/g xylitol yield. Finally, they successfully crystallized xylitol from CG/xylose and PG/xylose fermented broths with recovery of 35.3 and 39.5%, respectively. This study demonstrated the potential of *Y. lipolytica* as a microbial cell factory for the production of xylitol from low-cost feedstocks (Prabhu et al., 2020).

### Isomaltulose

Isomaltulose (IM) is a disaccharide sugar usually present in nature. It consists of glucose and fructose monomers which are connected with each other through α-1,6 glycosidic linkage (Shyam et al., 2018). The well-known trade name of IM is Palatinose^TM^. IM is found to be a stable but completely digestible sugar with low glycemic index (Maresch et al., 2017). IM garnered attentions as an alternative sweetener to sucrose. IM is also involved in management and prevention of chronic diseases like cancers and cardio-metabolic diseases (Shyam et al., 2018). IM is mostly produced from sucrose by sucrose isomerase (SIase) (Zhang et al., 2018). By overexpressing SIase from *Pantoea dispersa* UQ68J in *Y. lipolytica* and converting monosaccharide byproducts into intracellular lipids, Zhang et al. (2018) produced 572.1 g/L IM with 97.8% purity in a final fermented broth. To circumvent the obstacle of cell membranes, the Zixin Deng group displayed a SIase on the *Y. lipolytica* cell surface by using the cell wall protein (CWP) Pir1 and achieved 465 g/L IM production. The cell surface displayed that SIase was found to be stable under broad ranges of temperature (20–40°C) and pH values (4.5–7.0) and maintained more than 80% activity after 12 reaction cycles (Li et al., 2017). Zheng et al. (2019) also displayed a SIase on the *Y. lipolytica* cell surface by utilizing the glycosylphosphatidylinositol (GPI)-linked CWP anchor signal sequence. The cell surface display achieved the highest SIase activity of 2,910.3 U/g of dry cell weight. The surface displayed SIase showed the same optimal temperature (30°C) as the free SIase, but higher thermostability and longer half-life than the free SIase. In the subsequent IM production from low-cost cane molasses, the IM conversion rate maintained more than 85% after nine cycles of reaction. The high operational stability is a desired characteristic in industrial production (Zheng et al., 2019).

### Trehalose

Trehalose is a non-reducing disaccharide composed of two glucose subunits connected by α,α-1,1-glycosidic linkage. Because of the outstanding osmoprotective activity, trehalose serves as one of the most efficient functional molecules to protect the cells from stresses like drought, salt, and heat (Li et al., 2009; Trevisol et al., 2011). Trehalose has also been used as a neuroprotective agent against neurodegenerative diseases like Huntington and Parkinson in animal models (Lee et al., 2018). In *S. cerevisiae*, trehalose synthesis involves two synthetic enzymes trehalose-phosphate synthase (Tps1p) and trehalose-6-phosphate phosphatase (Tps2p), and two regulatory proteins Tps1p and Tps3p (Reilly and Doering, 2010). However, this pathway has not been explored for industrial scale synthesis. Trehalose is currently produced by two enzyme-based processes. The first one is a multi-enzyme process, which utilizes maltooligosaccharides or starch as substrate and involves maltooligosyltrehalose hydrolase, maltooligosyltrehalose synthase, α-amylase, and pullulanase. The second process utilizes trehalose synthase (TreS) to directly transform maltose into trehalose through intra-molecular transglycosylation. The TreS-catalyzed one-step transformation is the most explored process, because it is fast, simple, and less expensive (Zheng et al., 2015). The Zixin Deng group simplified the TreS-based process by integrating enzyme production, trehalose transformation, and ethanol removal in one pot (Li et al., 2016). They firstly displayed the *Picrophilus torridus* TreS on *Y. lipolytica* cell surface by fusing it with the *Y. lipolytica* cell wall anchoring protein YlPir1. The optimal pH and temperature were subsequently figured out, and 219 g/L trehalose was produced. The displayed enzymes were found more stable at optimal pH and temperature in comparison with free enzymes. *S. cerevisiae* was used to ferment the residual maltose and glucose into ethanol, which was subsequently removed by distillation. Finally, high-purity trehalose was easily obtained from the broth. This bioprocess represents an easier and low-cost access to trehalose (Li et al., 2016).

### Fructo-Oligosaccharides

Fructo-oligosaccharides (FOSs) are oligosaccharides consisting of β-2,1 fructosyl-fructose glycosidic linkage (Chen et al., 2016). The FOSs have many applications including gastrointestinal improvement, modulation of immune system, colon cancer protection, reduction of obesity linked disease, and helping in mineral uptake (Chen et al., 2016). Fructosyltransferase (FTase) can be used to transform sucrose into FOSs. Multiple FTase-based biotransformation processes have been developed, including the use of whole cells harboring native FTase, purified FTase, immobilized whole cells, and immobilized FTase (Bali et al., 2015). Although the immobilized methods are effective, the carriers are expensive, and the manipulations are complicated. The Zixin Deng group developed an in-expensive and industrially attractive biotransformation process by utilizing the erythritol industry yeast pastes to produce FOSs. They engineered the erythritol-producing *Y. lipolytica* into FOS-producing whole cell catalyst by displaying an FTase from *Aspergillus oryzae* on the cell surface. Under the optimal conditions, the whole-cell catalyst produced 480 g/L FOS from 800 g/L sucrose, with a yield of 60% and productivity of 160 g/(L⋅h). The engineered yeast pastes from erythritol industry was stable in FOS production, with only 10% FTase activity lost after 10 recycling number (Zhang et al., 2016). FOSs can also be produced by directly hydrolyzing inulin by the endo-inulinase (EC 3.2.1.7) (Singh et al., 2016). Inulin is an abundant renewable natural resource. Han et al. (2017) developed a two-stage bioprocess using recombinant *Y. lipolytica* strain Enop56 to produce FOSs from inulin. *Y. lipolytica* Enop56 was constructed by overexpressing an optimized endo-inulinase encoding gene from *Aspergillus niger*. This method produced 546.6 g/L FOSs from 600 g/L inulin in a 10-L bioreactor, with a yield of 0.91 g/L and productivity of 15.18 g/(L⋅h). Moreover, FOSs were the main hydrolysis products, with only 4.97% of total amount of non-prebiotic saccharides in the fermented broth (Han et al., 2017). This will greatly simplify the downstream purification process. This method also showed promising industrialization potential.

### Galacto-Oligosaccharides

Galacto-oligosaccharides (GOSs) are lactose-derived non-digestible prebiotics and are widely used as substitutes of human milk oligosaccharides in milk formulas of infants and newborn babies (Vera et al., 2016). GOSs have been produced from lactose by using free or immobilized β-galactosidase (EC 3.2.1.23), which shows both glycoside hydrolase and galactosyltransferase activities (Vera et al., 2016). However, these methods suffer from time-consuming, expensive carriers, enzyme diffusion, and/or loss of activity (Bilal et al., 2020). To resolve these issues, the Zixin Deng group displayed an *Aspergillus oryzae* β-galactosidase on the cell surface of the erythritol producing *Y. lipolytica*. The recombinant strain produced 160 g/L GOSs from 500 g/L lactose, with a yield of 51% of consumed lactose. The optimal temperature of the surface displayed β-galactosidase was found to be 20°C higher than that of the free enzyme. The surface displayed β-galactosidase showed substantial stability during the GOS production, in which GOS yield did not decrease significantly even after 10 rounds of reaction (An et al., 2016).

## Gene Discovery and Pathway Engineering

*Yarrowia lipolytica* has the potential to produce erythritol at high level from glycerol. However, it also consumes erythritol as carbon source, which has negative impact on erythritol production. By using insertional mutagenesis strategy, Carly et al. (2017) obtained a mutant without erythritol catabolism. Subsequent genome sequencing confirmed that the mutant phenotype is directly linked to the disruption of gene *YALI0F01606g*, which was suggested to rename as *EYK1*, encoding an erythrulose kinase. Their results also demonstrated that disrupting *EYK1* enhanced erythritol production from glycerol (Carly et al., 2017). In another study, they identified and characterized another erythritol metabolism gene *EYD1*, encoding the erythritol dehydrogenase in *Y. lipolytica*. They found that strains containing disrupted *EYD1* cannot utilize erythritol as carbon source. They further used EYD1 for the bioconversion of erythritol into erythrulose. By constitutively expressing *EYD1* in an *EYK1* disrupted *Y. lipolytica* chassis, erythrulose was produced at a rate and yield of 0.116 g/(g_*DCW*_⋅h) and 0.64 g/g (Carly et al., 2018).

Recently, the erythritol metabolic pathway in *Y. lipolytica* was characterized by functionally overexpressing four genes involved in the pentose phosphate pathway. Among them, the *TKL1* (*YALI0E06479g*, encoding transketolase) was found to be a crucial gene for the erythritol synthesis. Overexpressing *TKL1* improved erythritol titer by twofold in shaking flasks and 70% in 5-L bioreactor at low agitation. In addition, overexpressing *TKL1* permits efficient erythritol production at low dissolved oxygen level (Mironczuk et al., 2017).

The NAD(P)H dependent erythrose reductase (ER) catalyzes the final and crucial step of erythritol synthetic pathway in *Y. lipolytica*. Janek et al. validated a predicted native ER encoding gene (*YALI0F18590g*) by overexpressing and characterizing its influence on erythritol synthesis in *Y. lipolytica*. The results showed that overexpressing *YALI0F18590g* improved erythritol titer by 20%, and Zn^2+^ had a positive effect on the activity (Janek et al., 2017). Later, Cheng et al. identified another two novel erythrose reductases (ER25 and ER10) by characterizing the purified enzymes and overexpressing the respective encoding genes in *Y. lipolytica*. Finally, by overexpressing the newly isolated ER genes and engineering NADPH metabolism, they produced 190 g/L erythritol in baffled flasks, with the yield and productivity improved by 23.5 and 50%, respectively (Cheng et al., 2018).

*Yarrowia lipolytica* accumulates the osmoprotectant erythritol as a response to the hyperosmotic stress. The high osmolality glycerol (HOG) pathway, which has been well studied in *S. cerevisiae*, senses and responds to the stressful hyperosmotic signals. Rzechonek et al. (2018) identified a *Y. lipolytica* homolog of HOG1 (yl-Hog1, encoded by *YALI0E25135g*) and proved that it is responsible for the response to the hyperosmotic stress and induction of erythritol production.

## Expanding Substrate Scope and Bioprocess Engineering

Expanding the substrate scope to inexpensive materials or industrial wastes is helpful to improve the economical and environmental effects of sugar alcohol and functional sugar production. Crude glycerol is a main byproduct of the biodiesel industry and is a cheap and renewable resource. Rakicka et al. (2016) developed a chemostat culture process and obtained very promising results when they replaced pure glycerol with crude glycerol (103 vs. 81.9 g/L) as carbon source. Several other studies has been published on the production of erythritol from crude glycerol in recent years (Mironczuk et al., 2016; Yang et al., 2016; Rakicka et al.,2017a,b; Da Silva et al., 2018; Rakicka-Pustulka et al., 2020).

Okara (soybean residue), oil crop wastes, and waste cooking oil (WCO) have also been used for erythritol production. Okara is thought to be an ideal substrate for its low-price and high-nutrient content. Liu et al. developed, optimized, and scaled up a pretreatment and fermentation process using okara as feedstock. No mineral or nitrogen supplementation was added during the erythritol producing process (Liu et al., 2017b). However, to make okara more accessible, hydrolysis by fungal in-house enzymes was needed before erythritol production. This will make the process more complicated and time-consuming (optimally 5-day pretreatment).

Oil crop wastes are attractive and economical feedstocks for erythritol production in *Y. lipolytica*. However, undesirable excessive nitrogen content in oil crop wastes hampers the erythritol yield, whose synthesis is triggered only under nitrogen starvation condition. To resolve this issue, Liu et al. deleted the sucrose non-fermenting protein kinase gene (*SNF1*), which is involved in the nitrogen starvation-triggered process. The carbon source utilization and erythritol production were enhanced by the engineered strain under nitrogen-rich conditions (Liu et al., 2020). In the following research, they developed a one-step solid-state fermentation process, in which improved erythritol production was achieved from unrefined oil crop wastes using this *SNF1*-deficient *Y. lipolytica*. The developed process showed advantages of lower material cost and shorter fermentation period (Liu et al., 2019a). Their studies represent a new way for the development of cost-effective method for the effective synthesis of *Y. lipolytica* metabolites from nitrogen-rich wastes.

The WCO from catering services can no longer be reused because of the oxidized fatty acids and hazardous components. However, the high energy value and low-price make WCO an excellent alternative carbon source for the oleaginous yeast *Y. lipolytica*. Liu et al. investigated the crucial factors of osmotic pressure and pH in the erythritol and citric acid production from WCO. Their results showed that high osmotic pressure together with low pH promoted erythritol production and inhibited citric acid production, and vice versa (Liu et al., 2018). In another study, they used an agricultural waste loofah sponge (LS) as dispersant to improve the insolubility of WCO in the culture. The results showed that LS enhanced the WCO dispersion, utilization, *Y. lipolytica* growth, and erythritol production. LS was stable during cultivation process and can be easily recycled (Liu et al., 2019b).

## Breeding Efficient Strains Using Novel Mutation Method

**FIGURE 2:**
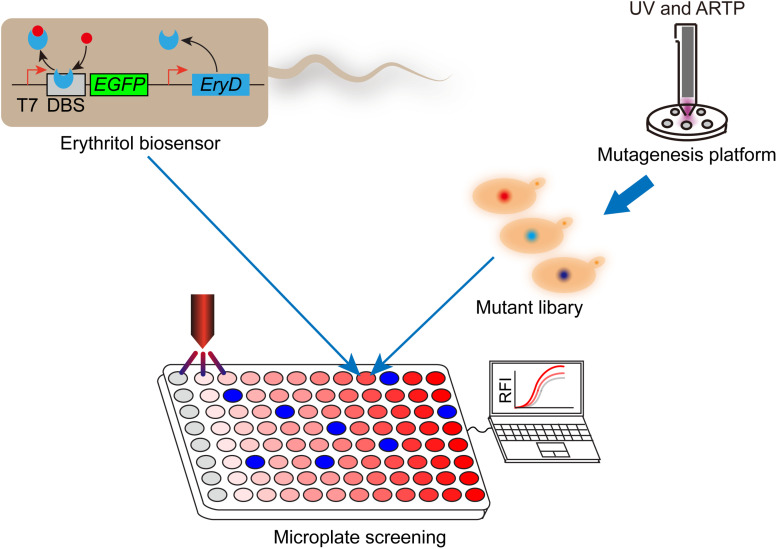
Work flow of combining genetically encoded erythritol biosensor with high-throughput screening to improve erythritol production in *Y. lipolytica*. The erythritol biosensor was developed in *Escherichia coli* BL21 (DE3). Transcriptional repressor EryD and cognate DNA-binding site were used to control the expression of enhanced green fluorescence protein (GEFP). After mutagenesis, mutant library of erythritol producing *Y. lipolytica* was combined with biosensor and submitted for microplate-based high-throughput screening. High performers can be screened out according to the fluorescent signals. T7, T7 promoter; DBS, EryD DNA-binding site; RFI, relative fluorescence intensity.

In addition to genetic engineering and bioprocess engineering, breeding competitive strains are an important alternative approach to improving sugar alcohol production in *Y. lipolytica*. The traditional mutation processes are usually not environmentally friendly. The atmospheric and room temperature plasma (ARTP) is an emerging mutation platform, whose process is rapid, low cost, under low temperature, and environmentally friendly (Liu et al., 2017a). In this section, we will discuss the recent studies about breeding efficient sugar alcohol producing strains by using ARTP platform. Using ARTP platform, Liu et al. established a mutant library of marine fish gut isolated *Y. lipolytica*, which was found to be able to accumulate erythritol in their previous study. The best-performing mutant produced 169.3 g/L erythritol in 168 h in a 5-L fermentor in fed-batch fermentation, while low-level byproducts were detected in the broth (Liu et al., 2017a). Although the ARTP platform can produce large-scale mutant library conveniently, screening the best-performing mutant out from the library is laborious and time-consuming. Qiu et al. developed a genetically encoded biosensor-regular platform to rapidly screen and characterize the erythritol overproducers from the mutant library (Figure 2). The transcriptional repressor EryD and cognate DNA-binding sequence were utilized to control the expression of a fluorescence reporter (eGFP). In the absence of erythritol, the *EGFP* transcription was repressed; while in the presence of erythritol, the transcriptional repression was relieved. By coupling this biosensor-regulator system with microplate reader, they screened and characterized a library of more than 1,152 mutants derived from combined UV and ARTP mutagenesis, in a short period of time (1 week). The best-performing mutant produced 148 g/L erythritol in bench-top fermenter. By coupling genetically encoded biosensor with mutagenesis, this study provided a convenient high-throughput screening and characterization framework to improve the performance of industrial microbial producer (Qiu et al., 2020).

## Author Contributions

YL and JX conceived the topic. AA and YL drafted the manuscript. YL and JX revised the manuscript. JL, ZW, AZ, HY, LQ, MA, and WX gave suggestions. All authors contributed to the article and approved the submitted version.

## Conflict of Interest

JX was also employed by companies Zhengzhou Tuoyang Industrial Co., Ltd and Zhengzhou University Industrial Technology Research Institute Co., Ltd. The remaining authors declare that the research was conducted in the absence of any commercial or financial relationships that could be construed as a potential conflict of interest.
